# Critical current fluctuations in graphene Josephson junctions

**DOI:** 10.1038/s41598-021-99398-3

**Published:** 2021-10-06

**Authors:** Mohammad T. Haque, Marco Will, Matti Tomi, Preeti Pandey, Manohar Kumar, Felix Schmidt, Kenji Watanabe, Takashi Taniguchi, Romain Danneau, Gary Steele, Pertti Hakonen

**Affiliations:** 1 Low Temperature Laboratory, QTF Centre of Excellence, Department of Applied Physics, Aalto University School of Science, P.O. Box 15100, 00076 Aalto, Finland; 2 Kavli Institute of NanoScience, Delft University of Technology, Lorentzweg 1, 2628 CJ Delft, The Netherlands; 3 Research Center for Functional Materials, National Institute for Materials Science, 1-1 Namiki, Tsukuba, 305-0044 Japan; 4 International Center for Materials Nanoarchitectonics, National Institute for Materials Science, 1-1 Namiki, Tsukuba, 305-0044 Japan; 5 Institute for Quantum Materials and Technologies, Karlsruhe Institute of Technology, 76021 Karlsruhe, Germany

**Keywords:** Condensed-matter physics, Nanoscience and technology, Graphene

## Abstract

We have studied 1/*f* noise in critical current $I_c$ in h-BN encapsulated monolayer graphene contacted by NbTiN electrodes. The sample is close to diffusive limit and the switching supercurrent with hysteresis at Dirac point amounts to $\simeq 5$ nA. The low frequency noise in the superconducting state is measured by tracking the variation in magnitude and phase of a reflection carrier signal $v_{rf}$ at 600–650 MHz. We find 1/*f* critical current fluctuations on the order of $\delta I_c/I_c \simeq 10^{-3}$ per unit band at 1 Hz. The noise power spectrum of critical current fluctuations $S_{I_c}$ measured near the Dirac point at large, sub-critical rf-carrier amplitudes obeys the law $S_{I_c}/{I{_c}}^2 = a/f^{\beta }$ where $a\simeq 4\times 10^{-6}$ and $\beta \simeq 1$ at $f > 0.1$ Hz. Our results point towards significant fluctuations in $I_c$ originating from variation of the proximity induced gap in the graphene junction.

## Introduction

The characteristic density of states at Fermi level with linear energy dependence in monolayer graphene^1,2^ influences fundamentally the electrical 1/*f* noise of graphene devices. Low frequency perturbations in the chemical potential will consequently lead to variation in resistance, giving rise to 1/*f* noise. Such current noise amounting to $\delta I/I \sim 10^{-4}$ per unit band (at 1 Hz)^3^, may originate, for example, from charge traps, localized carrier states at the edges, or fluctuations in the dielectric constant of the gate insulator, leading to involved gate voltage dependence of 1/*f* noise^4–12^. Part of the 1/*f* noise originates from charge carrier mobility fluctuations which may arise a.o. due to substrate roughness, ripples in graphene, gas adsorbates, or coupling to phonons^2,7,13–15^. In h-BN encapsulated graphene samples, a weak gate modulation in the current fluctuation is seen with peak at Dirac point and saturation at higher charge density^16,17^. This reduction in noise is inherent to reduced charge density fluctuations in h-BN encapsulated devices^18,19^. In such samples, contact resistance is prone to low-frequency fluctuations^9,10^. Recently, it has been observed that current crowding^20,21^ plays an important role in 1/*f* noise at graphene-metal contacts^22^.

In proximity-induced superconducting graphene junctions (SGS)^23^, the 1/*f* noise mechanisms are expected to be the same as in non-superconducting junctions. However, if the fluctuations are conveyed into fluctuations in the pair correlation length, then even stronger 1/*f* noise may appear in the critical current $I_c$ of SGS junctions^24^. In short ballistic SGS junctions fabricated using h-BN encapsulation, the critical current has been demonstrated to grow with the number of transmission channels^25–28^. This strong tunability of ballistic SGS junctions makes them as a unique platform for realizing quantum circuits with long-lived coherent states. Microwave spectroscopy on short ballistic SGS Josephson junctions has demonstrated resilience of these junctions against strong magnetic fields, which further enhances their promise for hybrid quantum circuits^29–31^. It has been known for quite some time that the low frequency fluctuations are detrimental for the coherent operation of superconducting qubits^32^. Despite the importance of critical current fluctuations in SGS junctions for their coherence properties and, for instance, for the sensitivity of novel graphene bolometers^33,34^, detailed study of low frequency $I_c$ fluctuations in such junctions is still missing. Here we report detailed study of critical current fluctuations in h-BN encapsulated graphene proximity Josephson junctions.

Theoretical models for 1/*f* noise in graphene devices are basically models that utilize the same phenomenological approaches developed for semiconductor devices^35–37^. Correlation between mobility and carrier number density (*n*) fluctuations^38^ is expected to take place in graphene according to several models and it has indeed been observed in graphene experiments^6,12^. In graphene, short and long range scatterers have distinct roles, which can produce quite different charge density dependencies for 1/*f* noise in different samples^7,8^. The majority of scatterers may also reside in charge traps, which naturally leads to correlation between mobility fluctuations and carrier number^39^. In small devices, charge noise acting on the gate-sensitive channel resistance of graphene has also been employed to account accurately for low frequency current noise^5^. In Ref.^40^, charge traps have been considered in the context of SGS junctions and $<I_c(t) I_c(0)>$ noise correlators have been calculated in terms of time-dependent chemical potential fluctuations. As in Ref.^5^, the central factor is the derivative of the conductance with respect to chemical potential, which gives distinct gate dependence for the variance $\delta I_c^2$ with the possibility of having a minimum of noise at the Dirac point.

In this work we use h-BN encapsulated graphene samples with NbTiN contacts to address 1/*f* noise in the magnitude of supercurrent induced by superconducting proximity effect in graphene. We employ microwave frequency reflection measurements at $600 - 650$ MHz to track the time-variation in magnitude and phase of a reflected signal carrier, the fluctuations of which are Fourier transformed to yield the fluctuations of the Josephson inductance $L_J$ of the SGS junction. Since the critical current $I_c \propto 1/L_J$, fluctuations of $L_J$ yield fluctuations of critical current $\delta I_c$ as $\delta I_c/I_c = -\delta L_J/L_J$. We find 1/*f* dependence for $I_c$ fluctuations at $f \ge 0.1$ Hz on the order of $\delta I_c/I_c \simeq 10^{-3}$ at 1 Hz. Analogous to earlier normal-state experiments^6,7^, we find a local noise minimum in the normalized critical current noise $S_{I_c}/{I_c^2}$ in the regime of residual charge density near the Dirac point. However, the obtained $S_{I_c}/{I_c^2}$ depends unexpectedly strongly on the gate voltage, and it increases monotonically with the gate-induced carrier number $n_g$ at densities $|n_g| < 2.3 \times 10^{10}$ cm$^{-2}$. The increase in $S_{I_c}/{I_c^2}$ with $|n_g|$ points towards enhanced inverse proximity effects with growing charge density, which boosts fluctuations in the proximity-induced gap in the SGS junction due to its small edge contact regions.

## Methods

### Samples

**Figure 1:**
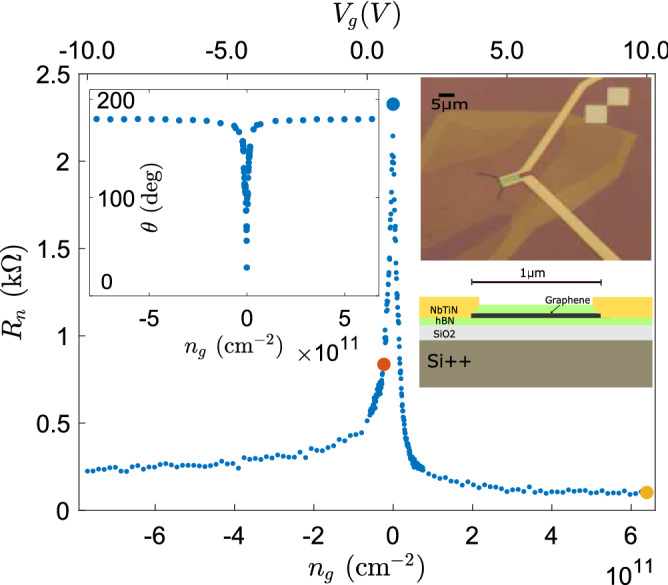
Zero-bias resistance $R_n$ versus gate-induced charge carrier density $n_g$ measured in the normal state at temperature $T=15$ K. Larger dots denote points at which low frequency noise of the SGS junction was studied in detail: (1) $n_g= -2.3\times 10^{10}$ cm$^{-2}$ ($V_g=0.6$ V, red), (2) charge neutrality point ($V_g^D=0.91$ V, blue) and (3) $n_g= 6.4\times 10^{11}$ cm$^{-2}$ ($V_g=10$ V, yellow). Left inset: Reflection phase $\theta$ versus gate-induced charge carrier density $n_g$. Right inset: Optical microscope image and cross-section schematic of the investigated two-lead sample: the width of the junction is 5$\,\mu$m while the length amounts to 1 $\mu$m. The yellow leads denote the NbTiN contacts. A 5 $\mu$m scale bar is indicated in the microscope image.

Our experiments employed encapsulated graphene Josephson junctions fabricated on strongly doped silicon with 285 nm of thermally grown oxide. The doped silicon acted as a back gate giving an areal capacitance of $C_{A} \simeq 1.13 \times 10^{-4}$ Fm$^{-2}$ for graphene on top of a 20-nm thick h-BN flake. NbTiN was selected as the contact material for its prospects for high upper critical magnetic field $B_{c_2}$^41^. The metallic leads of the sample were reactively sputtered from NbTi target in $\text {N}_2$ atmosphere which yielded superconducting side contacts with a critical transition temperature of $T_c = 13.4$ K. According to the BCS theory, this $T_c$ is equivalent to an energy gap of $\Delta \simeq 2$ meV. Such a large gap is beneficial for increasing the magnitude of the critical supercurrent. The charge density in the sample is tuned via Si$^{++}$ back gate. An optical microscope image and cross-section schematic of our primary sample is given in the inset of Fig. 1.

The main frame of Fig. 1 displays the normal state resistance $$R_n$$ of the graphene sample as a function of gate-induced charge carrier density $$ n_g$$ (gate voltage $$V_g$$). The charge neutrality point (CNP) is located at $$V_g^D = 0.91$$ V, which means *p*-doping for our sample at the charge carrier density level of $$n_{p}= 6 \times 10^{10}$$ cm$$^{-2}$$. The aspect ratio of the sample (width $$W = 5\,\mu $$m and length $$L =1\,\mu $$m) corresponds to $$\simeq 5$$ squares in parallel which leads to a minimum normal-state resistance of $$R_n \sim 2 $$ k$$\Omega $$. We assign the large value of the conductivity at the Dirac point to inhomogeneity in the residual charge density $$n_0$$ of the sample. Using $$-\log R_n$$ versus $$\log n$$ data, we extract $$n_0 = 8 \times 10^{9}$$ cm$$^{-2}$$ for the residual charge density at the CNP. We studied low frequency noise in details at three gate bias points (denoted as large filled circles in Fig. 1): (1) At charge neutrality point (CNP, blue circle), (2) close to CNP (red circle) and (3) far away from CNP (yellow circle). Mean free path, *l* is related to mobility $$\mu $$ and sheet conductance, conductivity $$\sigma $$ by the semiclassical relation: $$\sigma = e n_g \mu = \frac{2e^2}{\hbar }(\sqrt{\pi n_g}l)$$^42^. By taking the contact resistance approximately equal to the resistance at gate bias far away from CNP, we estimate the mean free path $$l=0.07 \,\mu $$m and $$0.13 \,\mu $$m, respectively, at the red and blue circles in Fig. 1. By comparing *l* with our channel length $$L =1\,\mu $$m, we observe that the sample is close to the diffusive transport limit. Additionally, we estimate for the Thouless energy $$24\,\mu $$eV, and $$43\,\mu $$eV, respectively, at these two charge densities $$n_g= -2.3\times 10^{10}$$ cm$$^{-2}$$ and $$|n_g|\approx 8\times 10^{9}$$ cm$$^{-2}$$ (CNP).

### Measurements

**Figure 2:**
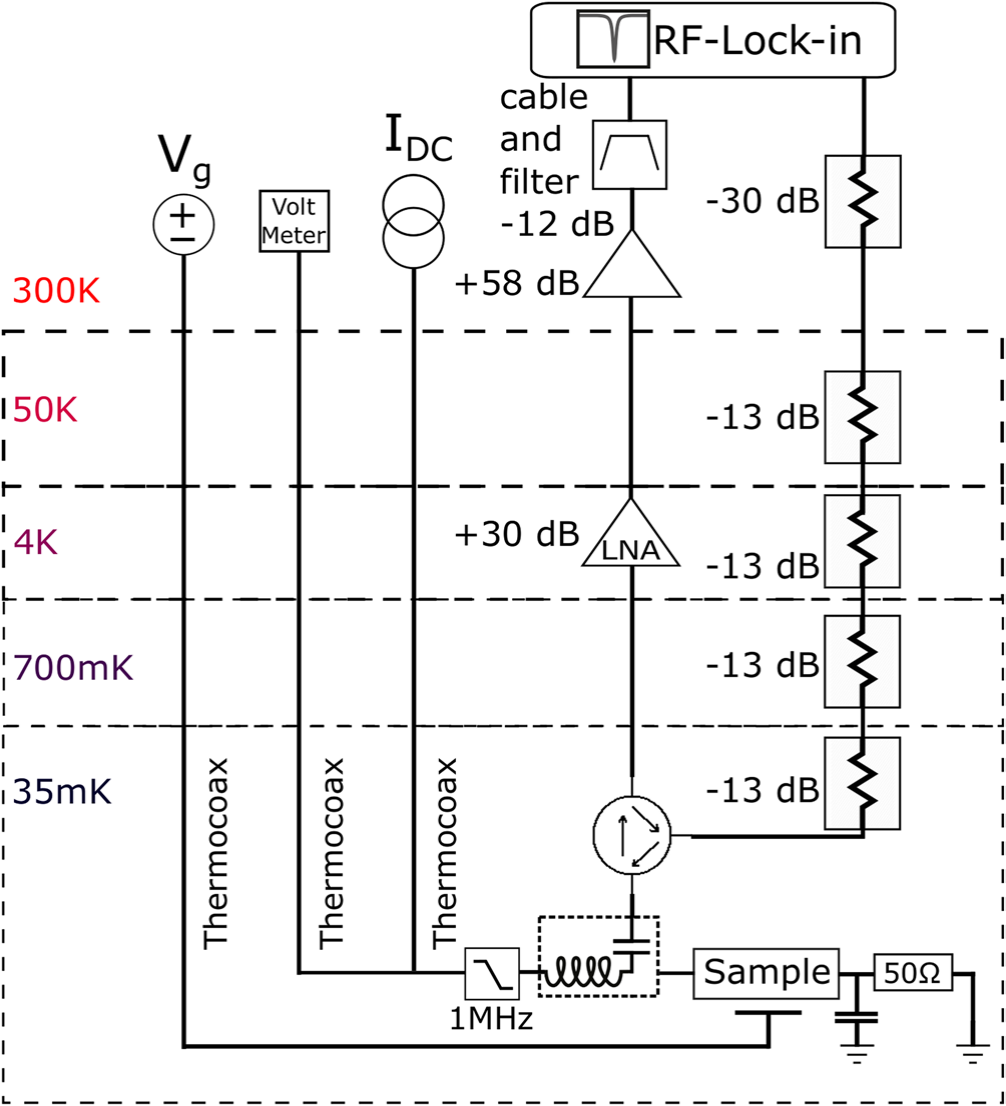
Schematics of the measurement setup. The audio (left half) and microwave (right half) circuitry is connected via a bias-T positioned between the sample and the circulator working over the band 600–900 MHz (Pamtech UTE1255K). The employed low noise amplifier was Caltech CITLF3. For details, see text.

The samples were mounted in a rf-tight copper enclosure with short $50\,\Omega$ transmission lines and SMA connectors. The experiments were performed on Bluefors LD400 dry dilution refrigerator with *T* = 10 mK base temperature. The electrical connections were via bias-T components (see Fig. 2) which allowed us to perform simultaneous low AC and microwave frequency measurements. The low frequency AC leads were equipped with *LC* and distributed *RC* filtering stages while the microwave line for reflection measurements had 53 dB of attenuation in total. The microwave port on the other bias-T was terminated to ground via a $50\,\Omega$ resistor. Ideally, if the impedance of the sample is zero, e.g. owing to a very large supercurrent, then the reflection becomes zero.

The low frequency noise was determined by tracking the sample impedance *Z* using reflection measurements and analyzing the fluctuations of the reflected voltage $v_m =\Gamma v_{rf}$ where $\Gamma =\frac{Z-Z_0}{Z+Z_0}$ is the reflection coefficient and $v_{rf}$ specifies the sent rf-carrier amplitude. Noise in amplitude and phase of $v_m$ was determined using an rf lock-in amplifier (Zurich Instruments, UHF 600 MHz) which also provided the rf-carrier signal at $f_{rf} = 600 - 650$ MHz. The lock-in time constant was set to $\tau =0.98$ ms which provides a low frequency measurement range up to $\sim 100$ Hz. The $I={\mathrm {Re}}({v_m})$ and $Q={\mathrm {Im}}({v_m})$ quadrature signals from the lock-in amplifier (LIA) were sampled at a rate of 858 samples per/s. Altogether, the time record length for Fourier analysis was $2^{16} =65536$ points, which yielded a frequency resolution of 13.1 mHz. The instrument provided also a complex Fourier transform $dI(\omega ) + idQ(\omega )$ of the time record $I-I_0+i(Q-Q_0)$, where $I_0$ and $Q_0$ denote time averages of the quadrature signals. The noise power referred to the employed frequency bin width was then obtained as $dI(\omega )^2+dQ(\omega )^2$. The high end of the spectrum (extending up to 429 Hz at the used sampling rate) was discarded as it was influenced by the lock-in time constant.

Calibration of the measurement setup was done separately for LIA and the measurement system. In the LIA there was an offset in the quadrature signals at low power levels. These were measured separately in reference measurements and subtracted away from the actual measurements. The reflection measurement setup was calibrated by Short, Open, Load method (SOL) by having the necessary reference impedances inside the cryostat and by connecting them via a microwave switch and equivalent cabling to the measurement system. The calibration, however, did not fully account for the employed bond wires, which leaves a small uncertainty of $\sim 0.5$ nH for the inductance values after the calibration.

The sample was excited using an rf-carrier signal at voltage levels from 0.045 $\mu$V$_{pp}$ to 14 $\mu$V$_{pp}$. The actual excitation was, however, influenced by the sample impedance and the components connected to it. The last attenuator on the mixing chamber before the sample was -13 dB which, together with 50 $\Omega$ termination resistance, yielded an effective load line resistance of $Z_L \simeq 80\,\Omega$. Hence, if the sample impedance in the superconducting state obeys $|Z_S| < Z_L$, our measurement can be considered as current bias, while in the normal state near Dirac point $|Z_S| \gg Z_L$, and our measurement is voltage biased. Note that voltage in this case equals $\sim 2v_{rf}$ calculated for a 50 $\Omega$ system. In our analysis, we employ values that refer to voltages at 50 $\Omega$ termination unless otherwise noted.

For the critical behavior of a Josephson junction, it makes a difference whether the rf-carrier voltage or current biases the sample. In the voltage bias regime, there is a critical voltage for the carrier amplitude that depends on the frequency according to the ac Josephson relation $d\varphi /dt=2\pi V /\Phi _0$ where $\Phi _0=h/2e$ denotes the superconducting flux quantum. For a frequency of 600 MHz the critical rf-voltage amplitude becomes $v_{rf}^c=2.2\,\mu$V$_{pp}$ when a maximum phase swing of $\pi$ is assumed. For current bias, the value of $I_c$ defines the threshold.

We model our sample as an inductance *L* that is in series with a termination resistance $R_T \simeq 50\,\Omega$, shunted by a stray capacitance *C* on the order of 2 pF due to 2-cm-long cabling before the 50 $\Omega$ termination. Thus, the reflection arises from an impedance of the form

1 $$\begin{aligned} Z=\frac{R_T}{1 + i\omega CR_T}+i\omega L \end{aligned}$$

and the reflection coefficient $\Gamma =\Gamma _0 e^{i\theta }$ becomes

2 $$\begin{aligned} \Gamma = \frac{Z/Z_0-1}{Z/Z_0+1} \simeq \frac{\tilde{R}_T(1-i \omega CR_T)+i \widetilde{\omega L} -1}{\tilde{R}_T(1-i\omega CR_T)+i \widetilde{\omega L} +1}, \end{aligned}$$

which is valid in the limit $\omega CR_T \ll 1$. Here tilde denotes impedance scaled with the transmission line impedance of $Z_0$, for example $\tilde{R}_T = R_T/Z_0$. From Eq. (2) we obtain a relation between the inductance *L* and the reflection phase $\theta$ given by

3 $$\begin{aligned} \tan (\theta )=\frac{\mathrm {Im}(\Gamma )}{\mathrm {Re}(\Gamma )}. \end{aligned}$$

First, Eq. (2) is fitted to the measured data to determine effective values for $\tilde{R}_T$ and $\omega CR_T$, after which we can solve for *L* and obtain the Josephson inductance as $L_J=L-L_0$ where $L_0$ denotes an inductance due to two bond wires ($\sim$ 3 nH each) which were not part of the SOL calibration. The critical current corresponding to the determined $L_J=\hbar /2eI_c$ is illustrated later in Fig. 6 in comparison with the relative 1/*f* noise magnitudes.

## Results

Initially, the samples were characterized around $T=15$ K by low frequency lock-in conductance measurements as a function of applied gate voltage using a 30.4-Hz sinusoidal signal with voltage amplitude $\sim 11\,\mu$V$_{pp}$. The applied gate voltage was converted into the gate-induced charge carrier density using $n_g = (V_g-V_g^D) C_g/Ae$, where $V_g^D=0.91$ V, $C_g =0.56$ fF, and graphene area $A=5.0$ $\mu$m$^2$. The mobility $\mu =1.5\times 10^5$ cm$^2$/Vs was determined from the maximum slope of $R_n^{-1}$ versus $V_g$ in Fig. 1 using $\mu = \Delta \sigma / \Delta ne$, where $\sigma =R_{\square }^{-1}$ stands for inverse square resistance $R_{\square }$.

**Figure 3:**
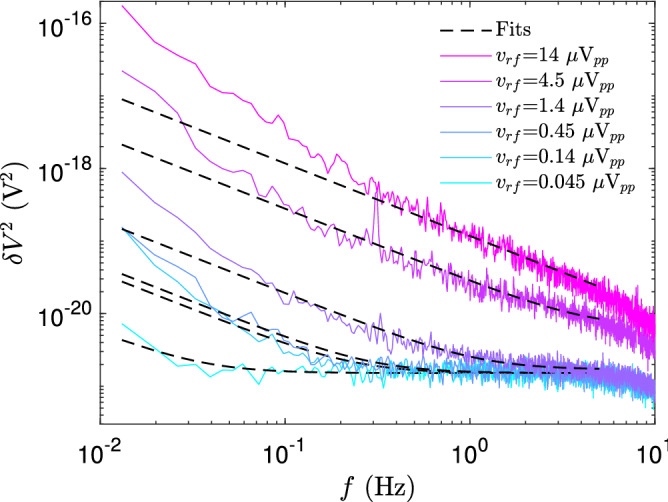
Noise spectra at $n_g= -2.3\times 10^{10}$ cm$^{-2}$ close to Dirac point in terms of squared voltage fluctuation $\delta V^2$ at the sample measured using rf carrier voltages specified in the inset. All of the noise spectra are measured at 35 mK temperature. The dashed lines and curves denote the 1/*f* fits with background noise taken into account as discussed in the text. In this measurement the number of points was $2^{17}$ which facilitates a minimum frequency of 13 mHz after omitting the lowest three points. The frequency bin for $\delta V^2$ corresponds to 3.28 mHz.

Figure 3 displays low frequency noise measured at $n_g = -2.3\times 10^{10}$ cm$^{-2}$ ($V_g = 0.6$ V) at a few rf carrier amplitudes denoted in the inset. All of the noise spectra are measured at 35 mK temperature. The small jump downwards in the noise power observed between $v_{rf} = 4.5\,\mu$V$_{pp}$ and 1.4 $\mu$V$_{pp}$ in Fig. 3 is identified as a crossover between normal and superconducting behavior. The noise at $v_{rf} = 4.5\,\mu$V$_{pp}$ carrier amplitude can be well split into two different regions: 1) $1/f^2$ noise behavior below 0.2 Hz, and 2) 1/*f* noise dependence above 0.2 Hz. At 1.4 $\mu$V$_{pp}$ and below, the system is in superconducting state and behaves as with current bias. In the superconducting regime, the noise power data still display a combination of $1/f^2$ and 1/*f* noise components, but the high frequency regime is dominated by the equivalent system noise voltage $v_n^2 \simeq 0.6\times 10^{-18}$ V$_{rms}^2$/Hz.

We are foremost interested in the 1/*f* part of the noise. Noise of the form $1/f^2$ is known to arise from single fluctuators, which are often present in small mesoscopic systems but they do not provide any universal qualities of the sample. The 1/*f* noise, on the other hand, is quite universal and can be employed as a general characteristics for evaluation of the sample quality. At high bias, the separation of the 1/*f* component by fitting is straightforward as the noise can be fitted directly by $\log (a/f)$ in the frequency range $f=0.2 - 40$ Hz using just a single parameter *a* (see the trace at $v_{rf}=4.5$ $\mu$V$_{pp}$ in Fig. 3). The roll-off around 50 Hz in Fig. 3 is caused by the selected lock-in time constant of 3 ms in these data.

At low bias, the noise power spectrum on logarithmic scale has been fitted using a function $\log \left( \sqrt{(a/f)^2+ b^2}\right)$ to a range of frequencies $\sim 0.05 - 0.5$ Hz, slightly adjusting the lower cut-off for avoiding contributions from the $1/f^2$ regime (see the data at $v_{rf}=0.14\,\mu$V$_{pp}$ in Fig. 3); here $b^2$ denotes the background noise $v_n^2$ over the frequency bin. At $v_{rf}=0.045$ $\mu$V, only the $1/f^2$ component is visible below 50 mHz. As a compromise, we have employed 0.1 Hz frequency to investigate how the noise power changes with carrier amplitude, in particular, when crossing from superconducting to normal region. We also measured high-bias current noise spectra directly at low frequencies in the normal state and verified that the spectra agreed within a factor of two with those measured using the carrier reflection method.

**Figure 4:**
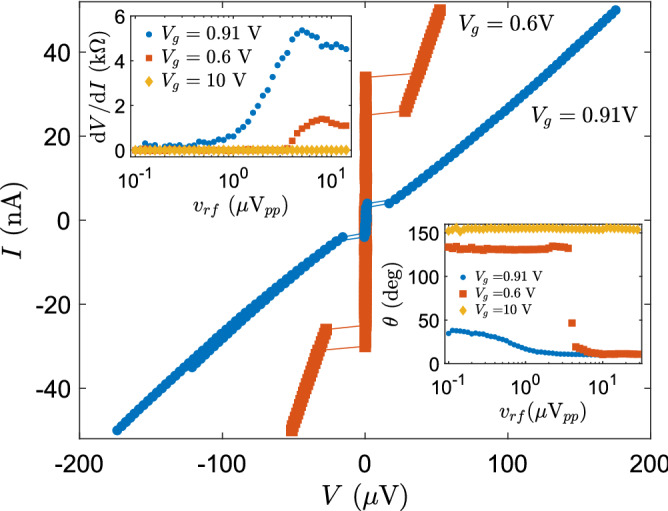
I–V curves measured at $V_g = 0.91$ V (CNP) and at $V_g = 0.6$ V ($n_g= -2.3\times 10^{10}$ cm$^{-2}$) at 35 mK temperature, while feeding simultaneously $v_{rf} = 0.33\,\mu$V$_{pp}$ on the sample from the oscillator of the lock-in amplifier. Right inset: Reflection phase $\theta$ versus carrier amplitude $v_{rf}$ measured at gate voltages indicated in the figure. Left inset: Differential resistance as a function of increasing $v_{rf}$ measured for three different gate voltages given in the figure.

Figure 4 displays the I–V characteristics at T = 35 mK measured at the CNP and near the CNP at $n_g= -2.3\times 10^{10}$ cm$^{-2}$ with rf carrier voltage $v_{rf} = 0.33\,\mu$V$_{pp}$ simultaneously applied. The I–V curves demonstrate superconductivity of the SGS junction with small hysteresis even in the presence of the rf carrier from the LIA. For the switching currents we obtain $I_{SW} \simeq 5$ nA and 35 nA at the Dirac point and at $n_g= -2.3\times 10^{10}$ cm$^{-2}$, respectively. An increase in $v_{rf}$ induces a gradual transition to the normal state at a gate-dependent carrier power. This is evident from the right inset of Fig. 4 which shows differential resistance *dV*/*dI* versus $v_{rf}$ for three different gate voltage values. At the Dirac point, the sample resistance begins to rise above zero around $v_{rf} \simeq 1\,\mu$V$_{pp}$ due to phase diffusion, while the sample becomes fully resistive at $v_{rf} \simeq 5\,\mu$V$_{pp}$. In comparison, for $n_g= -2.3\times 10^{10}$ cm$^{-2}$, the sample resistance starts rising later, around $v_{rf} = 3\,\mu$V$_{pp}$, and it crosses over to normal state at $v_{rf} \simeq 6\,\mu$V$_{pp}$.

**Figure 5:**
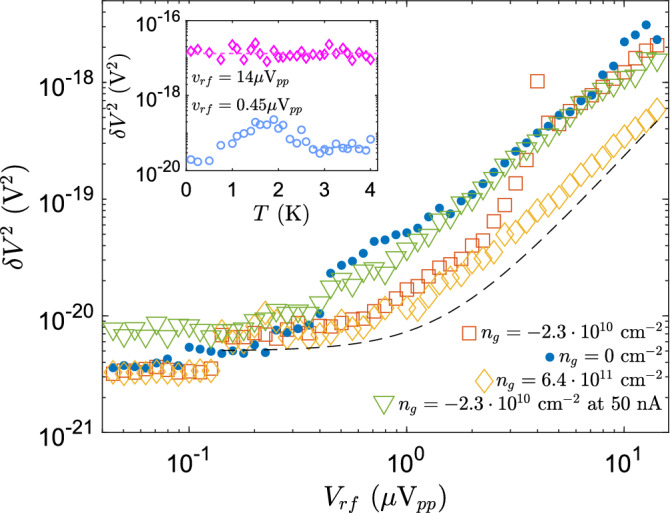
Noise power $$\delta V^2$$ using 13.1 mHz frequency bin at 0.1 Hz, taken from fitted curves illustrated in Fig. 3, as a function of carrier amplitude $$v_{rf}$$ for a few values of $$ n_g$$ indicated in the figure. At $$n_g= -2.3\times 10^{10}$$ cm$$^{-2}$$ we present also data with a DC bias current 50 nA $$>I_c$$ which forces the sample to normal state. The measurement gate voltage values are marked with respective color bullets on the normal state resistance versus carrier density plot, displayed in Fig. 1. The black dashed curve, representing $$a+b {\mathrm {V}}_{rf}^2$$, is a guide for the eyes. Inset: Change of noise power $$\delta V^2$$ at 0.1 Hz as a function of temperature at $$n_g= -2.3\times 10^{10}$$ cm$$^{-2}$$. Thermally induced phase diffusion in the variable $$\varphi $$ is seen as a bump in $$\delta V^2(T)$$ measured at $$V_{rf}^2=0.45\,\mu $$V$$_{pp}$$.

Figure 5 displays the measured noise power at 0.1 Hz as a function of rf-carrier voltage $v_{rf}$. Dependence of normal state noise on $v_{rf}$ is illustrated by the data under $I=50$ nA ($>I_c$) DC bias current, measured at $n_g= -2.3\times 10^{10}$ cm$^{-2}$. Strikingly, using line fits on log-log scale, the data displays $S_V \propto v_{rf}$ at $v_{rf} < 2\,\mu$V$_{pp}$, while the expected dependence $S_V \propto v_{rf}^{\gamma }$ with $\gamma \sim 2$ is reached at $v_{rf} > 2\,\mu$V$_{pp}$. Almost exactly the same relationship between noise and $v_{rf}$ is observed at the Dirac point without extra bias current. The noise in Josephson inductance tends to cause stronger fluctuations in the reflection signal when $\omega L_J \sim 2Z_0$ compared to the case $\omega L_J \ll Z_0$, which enhances the apparent noise power in the S state around the Dirac point in Fig. 5.

In the superconducting regime with $L_J$ small, the measured noise in the S state is clearly less than in the N regime as seen in Fig. 5 in the trace marked by ($\lozenge$), measured at $n_g= 6.4\times 10^{11}$ cm$^{-2}$. The noise is about $5-8$ dB lower in the superconducting regime than obtained in the normal state. This difference relates to a change in the effective impedance of the sample across the S-to-N transition. Ideally, the fluctuating voltage in the normal case is $\delta \Gamma v_{rf}\sim (2Z_0/Z)(\delta Z/Z)v_{rf}$ (when $|Z| \gg Z_0$), while in superconducting case the equation has a clearly different prefactor $\delta \Gamma v_{rf} \simeq ( \omega L/2Z_0)(\delta L/L) v_{rf}$ (far away from CNP where $\omega L \ll Z_0$). The $v_{rf}$ dependence in the superconducting case changes from linear to quadratic in the same fashion as observed in the N regime, illustrated in Fig. 5 by $(\nabla )$. The initially superconducting data at $n_g= -2.3\times 10^{10}$ cm$^{-2}$, however, is different in this respect as it displays an S-to-N transition with increasing carrier amplitude. The gradually increasing $v_{rf}$ dependence in the noise power makes it difficult to compare accurately the noise in the superconducting and normal states.

## Discussion

According to theory, the inverse Jospehson inductance is given by $1/L_J= (2\pi /\Phi _0)^2 \partial ^2E_J(\varphi )/\partial \varphi ^2$, where $E_J(\varphi )$ stands for the Josephson energy as a function of phase difference across the junction (see Ref.^43^ for graphene); for a tunnel junction (TJ) $E(\varphi )=-E_J \cos (\varphi )$. For illustrative purposes, we may consider a TJ for which $1/L_J= (2\pi /\Phi _0)^2 E_J \cos (\varphi )$. When the swing of $\varphi$ grows, either due to noise or experimental excitation, the mean value of $\cos (\varphi )$ is reduced and the effective Josephson inductance grows, till the Josephson energy is averaged out $\langle E_J(\varphi ) \rangle \rightarrow 0$ and the inductance $\langle L_J \rangle$ becomes infinity. According to Eq. (2), the reflection phase $\theta$ becomes smaller with increasing inductance. The gradual increase is clearly observed in the left inset of Fig. 4 near the Dirac point. The reflection phase decreases with increasing $v_{rf}$ and approximately zero is reached around $3.3\,\mu$V$_{pp}$ which corresponds to the critical voltage in the voltage biased case. At $n_g= 6.4\times 10^{11}$ cm$^{-2}$, a bias current of 1 $\mu$A is still below the critical current and the value of $\theta$ does not substantially decrease even at the strongest excitation. At $n_g= -2.3\times 10^{10}$ cm$^{-2}$, the destruction of the Josephson inductance at an rf current of $\sim 60$ nA$_{pp}$ is clearly observed. Note that this critical rf current is about the same as the DC switching current (*i.e.* $\simeq 2\times I_{sw}$) but three times smaller than $I_c$ deduced from the measured inductance.

**Figure 6:**
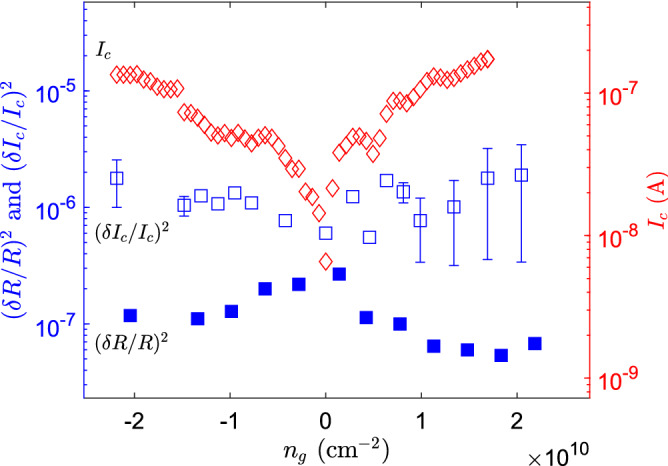
Left axis: Scaled critical current fluctuation $(\delta I_c/I_c)^2$ ($\square$), calculated from noise spectra measured at $v_{rf}=0.45-1.4\,\mu$V$_{pp}$. Scaled resistance fluctuation $(\delta R/R)^2$ ($\blacksquare$), calculated from noise spectra measured at $v_{rf}=14\,\mu$V$_{pp}$; for both variance traces the frequency bin is 13.1 mHz. The right axis gives the scale for the critical current marked by ($\lozenge$). In terms of Josephson inductance the range covers $L_J= 1.5 -40$ nH.

The measured noise in normal state was converted to resistance fluctuations using Eq. (2) by replacing $\widetilde{\omega L}$ with a resistive sample $\tilde{R}$. This yields $\delta R/R \simeq (R/2Z_0)\delta \Gamma$ where the measured $\Gamma$ is employed to determine the resistance of the sample *R* at microwave frequencies. When $R \gg Z_0$. For data at $v_{rf} > 5.6\,\mu$V$_{pp}$ we obtain per unit band $\delta R/R \sim 4 \times 10^{-3}$ at the Dirac point at 0.1 Hz, which corresponds to $\delta R/R \sim 4 \times 10^{-4}$ at 10 Hz. In Fig. 6 we display the measured weak gate dependence of $(\delta R/R)^2$ at 0.1 Hz. The normal state data do not display any increase of noise with growing $|V_g-V_g^D|$. This ”$\wedge$”-shape noise behavior is consistent with Hooge’s relation for disordered system and also inline with earlier reports on edge contacted samples^16^. Local modulation of the chemical potential close to the charge neutrality point will create in-homogeneous electron-hole charge puddles across the graphene sample. Variation of “percolating paths” among these charge puddles creates finite fluctuations of local resistance, giving rise to enhanced normalized current fluctuations around the Dirac point. Further away from the Dirac point, the sample resistance becomes dominated by the electrode-graphene interface and the contact-induced 1/*f* noise becomes dominant.

In the superconducting state, Josephson inductance fluctuations are dominated either by critical current fluctuations or by phase variation across the junction. At finite voltage, phase variation will be caused by phase diffusion due to phase slips, excited either thermally or by quantum tunneling^44^. The influence of thermally activated phase slips on the measured noise is illustrated in the inset of the Fig. 5: the measured voltage fluctuations $\delta V^2$ increase linearly with *T* above 400 mK. At temperatures $T > 2$ K, the graphene junction with $E_J/k_B \simeq 4$ K here switches to a fully running state *i*.e. normal resistive mode where, eventually, the voltage fluctuations are governed by the resistance noise.

Phase variation and inductance noise may also be induced by charge noise in quantum Josephson junctions in which charging energy becomes comparable to Josephson energy^45^. However, as our sample has good contacts, charging energy is not a relevant junction characteristics for it. According to Ref.^46^, in the limit of large scaled conductance $G/G_Q \gg 1$, the effective charging energy $E_{C_eff}$ for single electrons is exponentially renormalized as $E_{C_eff}/E_C \approx (G/G_Q) \exp (-\alpha G/G_Q)$ where $\alpha \sim 1$ is a dimensionless coefficient characterizing the material, $G_Q=2h/e^2$ is the conductance quantum and *G* is the conductance of the sample. From this relation, the effective charging energy at the CNP is on the order of 1 n*e*V which is negligible compared to Josephson energy $E_J$ $\sim$ 10 $\mu e$V.

In Fig. 5, the voltage fluctuations below a critical $v_{rf}$ amplitude reflect inductance variation (i.e. critical current fluctuations) in the reflection measurement. Since the I-V curves appear hysteretic in DC measurements, we believe that the junction phase becomes delocalized immediately above the critical rf amplitude (see the intermediate trace of $\theta$ in the inset of Fig. 4). Hence, the regime of critical current fluctuations transforms to resistive behavior without a phase diffusion branch in between, except for the vicinity of the CNP. This is illustrated in Fig. 5 by the data at $n_g= -2.3\times 10^{10}$ cm$^{-2}$ which indicates a steep change in the noise from S to N regime. Note that, at this crossover, the current induced by $v_{rf}$ will vary appreciably owing to voltage dependent impedance of the sample. On the contrary, data at $n_g= 6.4\times 10^{11}$ cm$^{-2}$ in Fig. 5 display only voltage fluctuations induced by inductive noise at a localized phase variable.

Even though the difference between S and N case in Fig. 5 is not very large, the difference becomes more apparent by the non-linear conversion of $\delta V^2$ to inductance fluctuation $\delta L_J$, arising from Eq. (2). The measured $S_{I_c}(V_g)$ shows a weak correlation with increasing $I_c(V_g)$. It is noteworthy that, for fixed inductance noise $\delta L_K={\mathrm {const}}.$, there would be an increase of $(\delta I_c/I_c)^2 \propto I_c^2$ with gate voltage. Thus, instead of external factors, variation in charge density in the sample is a more likely cause for the increase of the noise when moving away from the CNP.

Near the Dirac point, the normal state resistance noise $S_{R}/R^2$ agrees with the supercurrent noise $S_{I_c}/I_c^2$ within a factor of two. This is in line with the expectation based on the Ambegaokar-Baratoff (AB) relation $I_c R \simeq \Delta$. However, when moving away from the CNP, a growing deviation arises from the noise amount predicted by the AB-relation: $S_{R}/R^2 \sim S_{I_c}/I_c^2$. We assign the observed strengthening of $S_{I_c}/I_c^2$ with |*n*| to enhanced fluctuations due to variation in the proximity induced energy gap in the sample.

Small contacts on S-2D-S systems lead to strong modification of supercurrents. For example in the work of Kopnin et al.^47^, they showed that the AB relation becomes $I_c R_{eff} \simeq \epsilon _0$ where $R_{eff}$ is a weighted sum of graphene and contact resistance, and $\epsilon _0$ denotes the proximity induced gap. Consequently, supercurrent fluctuations are given by $S_{I_c}/I_c^2=S_{R_{eff}}/R_{eff}^2+ \delta \epsilon _0^2/\epsilon _0^2$. In particular, due to inverse proximity effect^47–49^, the latter term can vary substantially with charge density. The inverse proximity effect depends on the balance between the effective volume of the contact region (where the charge density is constant due to strong screening of the superconductor) and the charge density of the tuned 2D-material section. The effective contact length $\lambda$ depends on the interplay of these two regions. In general, when charge density in the graphene is increased, the effective $\lambda$ becomes smaller due to enhanced inverse proximity effect. The induced proximity gap is substantially suppressed in S-2D-S systems with $\lambda$ small compared with the 2D-material coherence length $\xi _{2D} \sim \sqrt{D/\Gamma _t}$; here *D* is the 2D diffusion constant and $\Gamma _t$ denotes the tunnelling rate (resistance) across the SN interface^47^. The reduced proximity gap is given by $\epsilon _0 \simeq \frac{\lambda L}{\xi _{2D}^2} \epsilon _{Th} \sim \Gamma _t \lambda /L$ where $\epsilon _{Th}=\hbar D/L^2$ stands for the Thouless energy. In the limit of weak contact transparency, $\Gamma _t$ coincides with the induced gap in the contact region, and thus we may approximate $\epsilon _0 \sim (\lambda /L)\Delta$ at high transparency. Approximating $L /\lambda \sim 200$ and $\Gamma _t \sim \Delta$, at the CNP, we estimate the coherence length and induced gap to be about 100 nm and 10 $\mu e$V, respectively, but these values should be taken only as order of magnitude estimates due to the very approximate estimate for $\Gamma _t$ and $\lambda$. The inverse proximity effect is also influenced via $\xi _{2D}^2$ due to charge traps at the contacts and at the graphene/BN interfaces as they influence $\Gamma _t$. Consequently, modifications in $\Gamma _t$ or changes in the effective $\lambda$ due to variation in the charge density can make a substantial change in $I_c$. Fluctuations of $\xi _{2D}^2$ and $\delta \lambda ^2$ are then seen in Fig. 6 as a weak growth of $(\delta I_c/I_c)^2$ when the charge density is increased, while $(\delta R/R)^2$ becomes simultaneously slightly reduced. The importance of contact properties for noise in normal graphene has been already been realized using suspended graphene^11^.

The influence of charge traps in superconducting graphene junctions has been investigated theoretically by Pellegrino *e*t al. who developed a chemical potential fluctuation model based on charge traps^40^. Qualitatively, our data for the superconducting state is in agreement with this theoretical model as the noise grows away from the Dirac point. With charge traps, however, the amount of noise should be related with the derivative $dG/dV_g$ which we don’t find in our experiments. Further experiments on samples with different contact structures will be needed to resolve whether SGS junctions with edge contacts are more susceptible to low frequency critical current noise than other SNS junctions.

## Conclusions

We have studied low-frequency fluctuations in Josephson inductance in h-BN encapsulated, monolayer graphene SGS junctions using microwave reflectometry at frequencies of $600-650$ MHz. We find $S_{I_c}/{I{_c}}^2 = a/f^{\beta }$ with $a\simeq 4\times 10^{-6}$ and $\beta \simeq 1$ at $f > 0.1$ Hz for the noise power spectrum of critical current fluctuations $S_{I_c}$ near the Dirac point. While scaled average fluctuation $\delta I_c/{I{_c}}$ corresponds nearly to the normal state resistance variation $\delta R/R$ at the Dirac point, a distinct difference by a factor of $\sim \sqrt{20}$ is observed at charge density $n_g= \pm 2 \times 10^{10}$ cm$^{-2}$ (see Fig. 6). We assign this increase in $\delta I_c/{I{_c}}$ to enhanced fluctuations in the proximity induced gap $\epsilon _0$, which governs the supercurrent in the graphene Josephson junction. The enhancement of the gap fluctuations $\delta \epsilon _0/\epsilon _0$ arises either from fluctuations in contact resistance $\Gamma _t$ or from the small nanometer-sized overlap length $\lambda$ of the edge contacts, for which $\delta \lambda /\lambda$ may become substantial due to small change in effective $\lambda$. Our work underscores contact quality as one of the central issues in future optimization of SGS junctions for superconducting quantum technology applications.

## Data Availability

The data that support the findings of this study are available from the corresponding author upon reasonable request.
